# Tissue Printing and Dual Excitation Flow Cytometry for Oxidative Stress—New Tools for Reactive Oxygen Species Research in Seed Biology

**DOI:** 10.3390/ijms21228656

**Published:** 2020-11-17

**Authors:** Danuta Cembrowska-Lech

**Affiliations:** Institute of Biology, University of Szczecin, Wąska 13, 71-415 Szczecin, Poland; danuta.cembrowska-lech@usz.edu.pl

**Keywords:** flow cytometry, germination, ImageJ, reactive oxygen species, tissue printing

## Abstract

The intracellular homeostasis of reactive oxygen species (ROS) and especially of superoxide anion and hydrogen peroxide participate in signaling cascades which dictate developmental processes and reactions to stresses. ROS are also biological molecules that play important roles in seed dormancy and germination. Because of their rapid reactivity, short half-life and low concentration, ROS are difficult to measure directly with high accuracy and precision. In presented work tissue printing method with image analysis and dual excitation flow cytometry (FCM) were developed for rapid detection and localization of O_2_^•−^ and H_2_O_2_ in different part of seed. Tissue printing and FCM detection of ROS showed that germination of wild oat seeds was associated with the accumulation of O_2_^•−^ and H_2_O_2_ in embryo (coleorhiza, radicle and scutellum), aleurone layer and coat. To verify if printing and FCM signals were specified, the detection of O_2_^•−^ and H_2_O_2_ in seeds incubated in presence of O_2_^•−^ generation inhibitor (DPI) or H_2_O_2_ scavenger (CAT) were examined. All results were a high level of agreement among the level of ROS derived from presented procedures with the ones created from spectrophotometric measured data. In view of the data obtained, tissue printing with image analysis and FCM are recommended as a simple and fast methods, which could help researchers to detection and level determination of ROS in the external and inner parts of the seeds.

## 1. Introduction

Reactive Oxygen Species (ROS) have long been known to be a damaging compound for biomolecules [1] and also signaling intermediates [2]. ROS are by—product of aerobic metabolism under natural conditions and plants have evolved an array of self-protective defensive tools to counteract loss of redox homeostasis of the cell [3,4]. Detoxification of ROS by antioxidative defense system comprising of both non-enzymatic and enzymatic antioxidants is paramount for the maintenance of physiological level of ROS and function of ROS as a signal molecules in a number of cellular processes.

Several works in the last decade strongly convey the role of ROS at all stages of seed life from embryogenesis to germination [5]. Out of several ROS, superoxide anion (O_2_^•−^), hydrogen peroxide (H_2_O_2_) and hydroxyl radical (HO•) have been implicated in dormancy releasing and germination [6,7,8]. ROS accumulation occurs both in quiescent dry seeds during after–ripening (AR) and metabolically active seeds [9,10]. ROS participate in dormancy release during AR through the direct oxidation of a subset of biomolecules, such as nucleic acid, especially mRNA [11]. During seed imbibition, their metabolic activity vary dramatically in most of the cell organelles like glyoxysomes, peroxisomes and mitochondria, results in ROS accumulation [12,13]. Likewise, the enzymatic activity of NADPH oxidase, which transfer electrons across the plasma membrane from cytoplasmic NADPH to molecular oxygen, is an important source of O_2_^•−^ in the plasma membrane [14]. Bailly et al. [5] proposed that the germination is completed only when the level of ROS is within ‘oxidative window for germination,’ which restricts the start of germination to a critical range of ROS level, enclosed by lower and higher thresholds. The ROS homeostasis is critical for seed germination and its concentration has influence on various signaling pathways, such as weakening of endosperm, seed reserve mobilization, protection against pathogens or programmed cell death (PCD) [13].

ROS are generated and scavenged over the whole life span of seeds. O_2_^•−^ has a relatively short half-life but it can migrate and can be used in the production of the more reactive and toxic HO• through the Haber-Weiss reaction [4,15,16]. H_2_O_2_ has a strong oxidizing capacity but its life span is longer than that of O_2_^•−^, it has the ability to cross membranes via aquaporins. Timing of generation, diffusion and degradation of various types of ROS within various cellular compartments leads to that ROS at different concentrations would play distinctly different roles therein, thus eventually leading to different fates of cells. Therefore, it has become very important to determine the location and concentration of ROS. Because ROS arise from various mechanisms, various qualitative and quantitative techniques are currently available. Furthermore, due to instability, short half-life and mutual interference of most oxygen radicals, ROS are not an easy object to analyze. Protocols for ROS detection and determination of its concentration in cell are available, like spectrophotometry [17], histochemical staining for stereomicroscopy and light microscopy [18,19], fluorescence [20,21], chemiluminescence [22], high-performance liquid chromatography (HPLC) [23], fluorescent protein-based redox probe [24], electron spin resonance (ESR) [25] and electrochemical biosensors [26]. However, each technique has its advantages and disadvantages, so it is recommended to use more than one method to test ROS in cells, trying to rule out specific drawbacks of single techniques [27].

Tissue printing onto membranes is a simple and rapid method employed to study the localization of nucleic acid, proteins and metabolites from freshly cut surface of a bisected tissue. Seed tissue imprints on nitrocellulose membranes were described by Reference [28] for localization of extensin in soybean seed coat. The KI/starch– or DAB–mediated tissue printing has been proposed for direct detection of H_2_O_2_ production in seedlings [29,30,31] and fruits [32]. A procedure based on the reduction of nitro blue tetrazolium (NBT) by O_2_^•−^, visualized by the formation of the dark blue formazan and oxidation of 3,3′-diaminobenzidine (DAB) by H_2_O_2_, visualized by the formation of the yellow complex, have recently been used for a localization of ROS in whole seeds or embryos isolated from seeds [33,34,35,36,37,38,39,40]. However, there is no evidence of studies using NBT- or DAB-mediated tissue printing for ROS analysis in seeds.

Detection and histochemical localization of ROS provides information about in situ distribution and accumulation of ROS in different part of tissue or organ. An important objective for analysis of stained tissue or tissue imprinting signal is to statistically compare staining intensity for a particular component that has been located in the analyzed object. The stained area can be detected and quantification analyzed with the methods of freeware or commercial applications for image analysis. The cost effective answer for quantitative analysis is ImageJ, image processing program inspired by NIH (National Institutes of Health) Image (https://imagej.nih.gov/ij/index.html). Image analysis with ImageJ is rapid and simply method, which proceeds as follows: acquisition of an image, preprocessing of the image for facilitating further processing, selection of pixels of interest and extraction of characteristic features. The quantitative analysis of O_2_^•−^ and H_2_O_2_ level based on histochemical staining was used for whole leaves [41,42,43]. However, there is no evidence of studies using method of quantification with the help of digital image analysis of histochemical staining and tissue printing results for ROS level estimation in seed.

Fluorescence is one of the excellent technique for visualize ROS due to the sensitivity and selectivity offered by fluorescent probe [44]. Detection of ROS by fluorescence involves the oxidation of the fluorescent probe itself but the probe being a stable molecule in the reduced state with fluorescence properties. Two specific dyes are available to measure intracellular ROS using fluorescence microscopy or standard fluorometer. Dihydroethidium (DHE) can detect intracellular O_2_^•−^ and 6-carboxy-2′,7′-dichlorodihydrofluorescein diacetate di(acetoxymethyl ester) (CDCDHFDA) are converted to fluorescent molecules in the presence of H_2_O_2_. DHE is very reactivity toward O_2_^•−^ and therefore widely used as a specific fluorescence probe for O_2_^•−^ [45,46]. CDCDHFDA is commonly used for detecting intracellular, subcellular and extracellular production of H_2_O_2_ to monitor redox changes in plant cells [47]. A probe fluorescence emission can be assessed by flow cytometry, which measures the fluorescence per cell. Flow cytometry (FCM) and fluorescence-activated cell sorting are widely used methods for the analysis of single animal cells and less frequently used with plant cells [48]. Until now, FCM has not been applied for measurement of ROS in seeds cells.

The aim of this work was to (1) adapt tissue printing methods for detection and localization of O_2_^•−^ and H_2_O_2_ in seeds, (2) use of ImageJ for estimation of ROS content for tissue printing signal and (3) use of flow cytometry for ROS level analysis. Application of tissue printing to detection of O_2_^•−^ and H_2_O_2_ in *Avena fatua* L. seeds is presented. To check whether the signal visible in the blotting membrane is associated with O_2_^•−^ and H_2_O_2_, signal was measured after treated of caryopses with DPI, an inhibitor of oxidase NADPH and CAT, an H_2_O_2_ scavenger. Content estimation of ROS was determined by an image processing software (ImageJ) as mean gray value of grayscale-converted NBT- or DAB-stained images, representing differences in staining intensity. ROS signal of NBT- or DAB-stained embryo isolated from caryopses incubated in presence of water, DPI or CAT solution was tested using ImageJ. Application of FCM to content estimation of ROS was also determined. The usefulness of tissue printing in ROS detection and localization in seed and ImageJ and FCM for relative estimation of ROS content in seed was explored using a spectrophotometric analysis.

## 2. Results

### 2.1. O_2_^•−^ and H_2_O_2_ Detection by Tissue Printing

**Figure 1:**
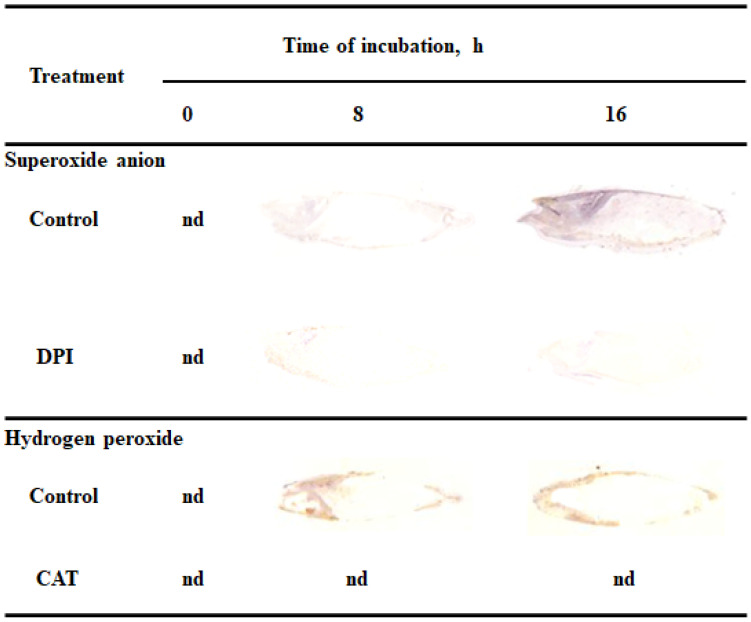
Detection and localization of O_2_^•−^ and H_2_O_2_ in *Avena fatua* seeds incubated in water, diphenyleneiodonium (DPI) (10^−4^ M) or catalase (CAT) (1000 U) at 20 °C for different time by NBT- or DAB-mediated tissue printing of seeds (representative tissue printing are shown). Nd-non detected.

Reactive oxygen species promote the germination of several seeds, both dormant and non-dormant [6,7]. In this study tissue printing was used to determine the detection and localization of ROS in non-dormant *Avena fatua* L. seeds during imbibition. Dry seeds and seeds incubated in water for 8 or 16 h were bisected and the cut surfaces were printed onto nitrocellulose membrane pre-soaked in NBT or DAB, immediately after cutting. To verify if printing signal was specified, were also examined the detection of O_2_^•−^ and H_2_O_2_ in caryopses incubated in presence of O_2_^•−^ generation inhibitor or H_2_O_2_ scavenger, DPI and CAT, respectively. O_2_^•−^ was not detected in dry seeds but seeds incubated in water for 8 h showed accumulation of O_2_^•−^ in embryo (in coleorhiza, radicle and scutellum), aleurone layer and coat (Figure 1). Interestingly, the coleoptile of the seeds were not stained. The intensity of the signals were increased in all parts of seeds and was more evident after 16 h, two hours before radicle protrusion through the coleorhiza. Moreover, the detection of O_2_^•−^ was suppressed when the seeds were incubated in presence of DPI for 8 and 16 h. In addition, to check whether the signal obtained is associated with mechanical damage, tissue printing was performed at 0, 1, 2 and 4 min after cutting. No significant differences were found for intensity of staining, when cut surface of a bisected seeds onto nitrocellulose membrane was pressed at 0, 1 and 2 min after cutting (data not shown) but after 4 min signal was strongly increased (Supplementary Materials Figure S1).

Presented data show that H_2_O_2_ was accumulated in embryo (in coleorhiza and radicle), aleurone layer and coat of seeds after incubation for 8 h but not in the scutellum and coleoptile (Figure 1). After this time the intensity of the staining increased up to 16 h of imbibition. Furthermore, in this time point, intensity of the staining in embryo was also detected in the scutellum but still not in coleoptile. However, when seeds were incubated in presence of CAT, intensity of printing signal was fully reduced. Similar to NBT-mediated tissue printing, no significant differences were found for intensity of staining, when cut surface of a bisected seeds onto nitrocellulose membrane, pre-soaked in DAB, was pressed only at 0, 1 and 2 min after cutting (data not shown).

### 2.2. In Situ Localization of O_2_^•−^ and H_2_O_2_ by Histochemical Staining

**Figure 2:**
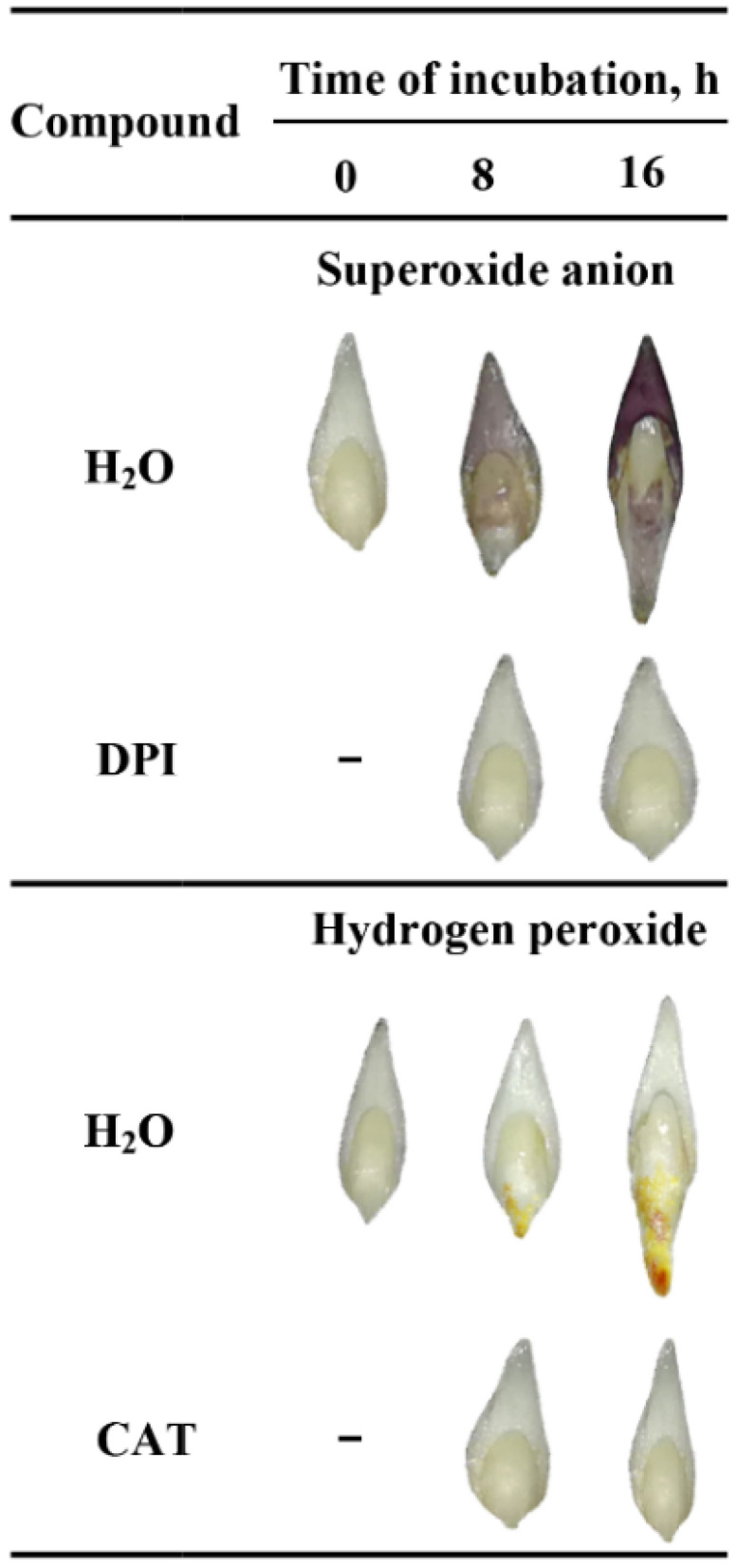
In situ localization of O_2_^•−^ and H_2_O_2_ in *A. fatua* embryos isolated from seeds incubated in water, DPI (10^−4^ M) or CAT (1000 U) at 20 °C for different time. Representative stained embryos are shown.

To verify the tissue printing results, the detection and localization of O_2_^•−^ and H_2_O_2_ were performed by histochemical NBT or DAB staining in the seeds imbibed in water, DPI or CAT. The experiment was first carried out using whole seeds but the staining was not completed; ROS were accumulating on the surface of coleorhiza of seeds when the coat was ruptured (Figure S2a–d). However, stained longitudinally bisected half caryopses showed accumulation of O_2_^•-^ and H_2_O_2_ in the coleorhiza, radicle, scutellum, aleurone layer and coat (Figure S3). The obtained data confirmed the results of tissue printing experiment (Figure 1). ROS were produced both on the outer and inner side of the cover. Furthermore, the signal intensity of stained longitudinally bisected half seeds was stronger than printing signal of half seeds. The tissue printing results shown, that ROS signal, not related with mechanical tissue damage, can be registered only when the tissue printing was done in less than 2 min after the cut was done. Probably, if the histochemical staining from NBT (last 10 min) or DAB (last up to 90 min) has been used to detect ROS in half seeds, resulted with very strong signal which might be related to mechanical damage. Therefore, the embryos were isolated from dry and imbibed seeds before NBT or DAB staining and it was found that embryos were stained (Figure 2).

In the embryo isolated from dry seeds accumulation of ROS was not detected (Figure 2). When seeds were incubated in presence of water for 8 h, embryo showed a significant accumulation of O_2_^•−^ and H_2_O_2_ in coleorhiza and scutellum or in coleorhiza, respectively, as compared to the same parts of the dry embryo. The intensity of the signal increased during imbibition time and was more evident after 16 h. However, when the seeds were incubated in presence of DPI or CAT, intensity of staining was fully decreased.

### 2.3. O_2_^•−^ and H_2_O_2_ Quantification by ImageJ

**Figure 3:**
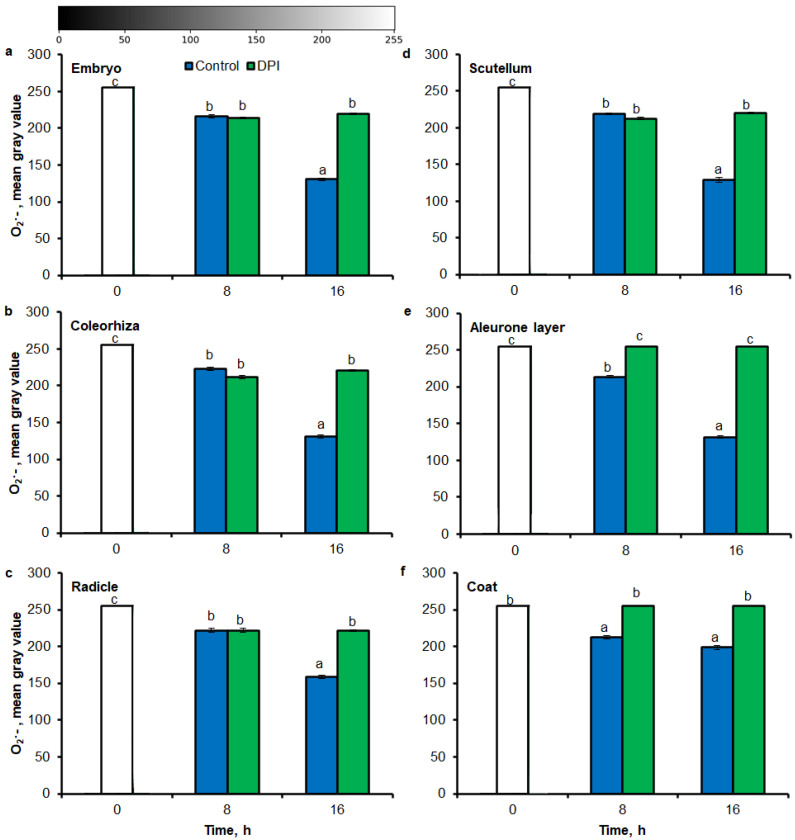
Quantification of O_2_^•−^ in *A. fatua* embryo (**a**), coleorhiza (**b**), radicle (**c**), scutellum (**d**), aleurone layer (**e**) and coat (**f**) of seeds incubated in water or DPI (10^−4^ M) at 20 °C for different time by image analysis (ImageJ) based on NBT-mediated tissue printing signal of seeds (see Figure 1). Mean gray value of grayscale converted NBT-stained images, representing difference in staining intensity. Grayscale intensities vary from 0 to 255 (0 = black, 255 = white) with the values in between representing shades of gray. Vertical bars indicate ± SD. One-way ANOVA with the Duncan’s *post hoc* test was used to determine the significance of differences. Mean values with different letters (a–c) are significantly different (*p* < 0.05, *n* = 5).

Digital image analysis package ImageJ was applied for quantification of the intensity of stained area in tissue printing from seeds and histochemical stained embryo. The quantification by the measure of staining intensity of printing signal showed that after incubation of seeds in presence of water for 8 h, the content of O_2_^•−^ was increased in embryo (Figure 3a), coleorhiza (Figure 3b), radicle (Figure 3c), scutellum (Figure 3d), aleurone layer (Figure 3e) and coat (Figure 3f) of seeds about 1.5 times in comparison to the same parts of dry seeds. The intensity of the signal increased during the imbibition and after 16 h content of O_2_^•−^ was about 2-fold higher compared with the dry seeds. The content of O_2_^•−^ in coat was similar over the time of imbibition. The detection of O_2_^•−^ was suppressed when the seeds were incubated in presence of DPI; the content of O_2_^•−^ has not changed throughout the whole period of imbibition.

**Figure 4:**
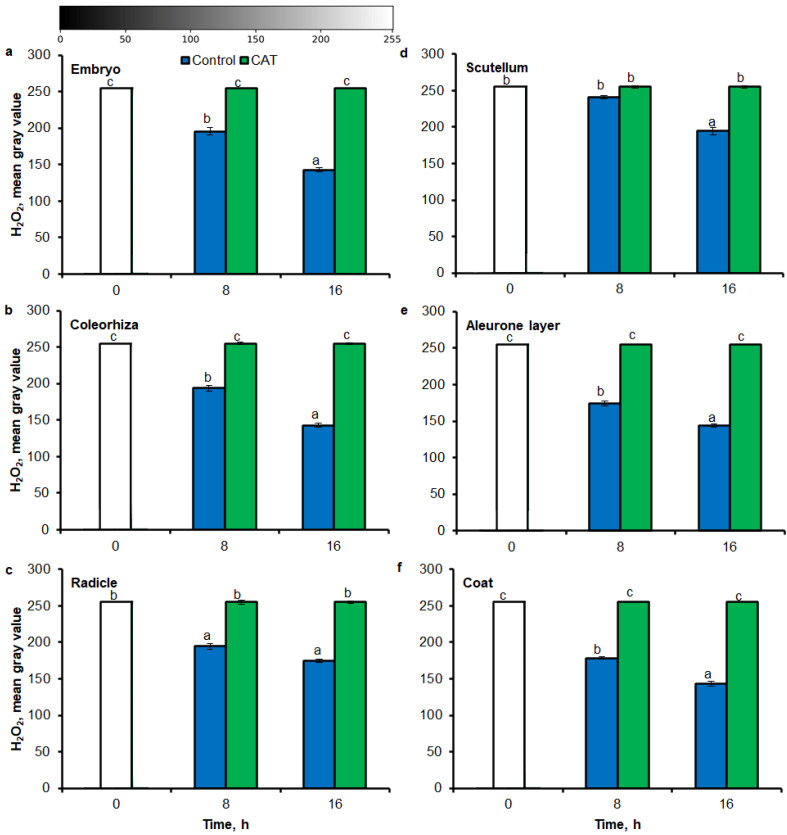
Quantification of H_2_O_2_ in *A. fatua* embryo (**a**), coleorhiza (**b**), radicle (**c**), scutellum (**d**), aleurone layer (**e**) and coat (**f**) of seeds incubated in water or CAT (1000 U) at 20 °C for different time by image analysis (ImageJ) based on DAB–mediated tissue printing signal of seeds (see Figure 1). Mean gray value of grayscale converted DAB–stained images, representing difference in staining intensity. Grayscale intensities vary from 0 to 255 (0 = black, 255 = white) with the values in between representing shades of gray. Vertical bars indicate ± SD. One–way ANOVA with the Duncan’s *post hoc* test was used to determine the significance of differences. Mean values with different letters (a–c) are significantly different (*p* < 0.05, *n* = 5).

Similar to results of NBT-mediated tissue printing, H_2_O_2_ was more strongly accumulated in embryo (Figure 4a), coleorhiza (Figure 4b), radicle (Figure 4c), aleurone layer (Figure 4e) and coat (Figure 4f) of seeds after incubation for 8 h in comparison to dry seeds; in all parts content of H_2_O_2_ was about 1.5-fold higher. Prolongation of incubation for up to 16 h progressively increased the intensity of the signal; the signal was ca. 1.8-fold higher in embryo, coleorhiza, radicle and aleurone layer from 16 h-imbibed seeds than signal from the dry seeds. Furthermore, in this time point, intensity of the staining in scutellum was only 1.3-fold higher than in scutellum from dry seeds (Figure 4d). The signal of H_2_O_2_ was not detected when the seeds were incubated in presence of CAT for 8 and 16 h.

**Figure 5:**
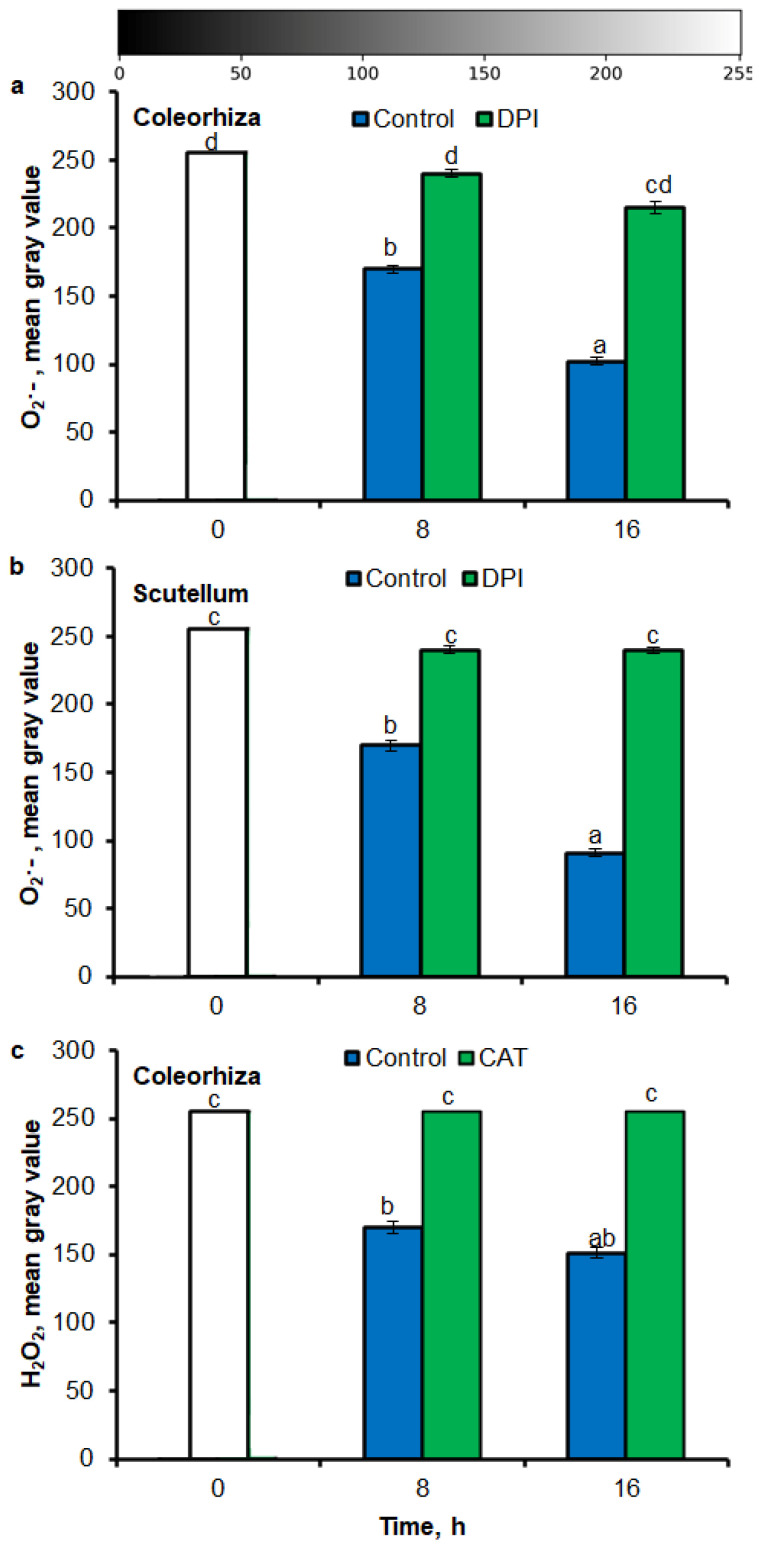
Quantification of O_2_^•−^ and H_2_O_2_ in *A. fatua* coleorhiza (**a**,**c**) and/or scutellum (**b**) of embryos isolated from seeds incubated in water, DPI (10^−4^ M) or CAT (1000 U) at 20 °C for different time by image analysis (ImageJ) based on stained embryo (see Figure 4). Mean gray value of grayscale converted NBT–stained images, representing difference in staining intensity. Grayscale intensities vary from 0 to 255 (0 = black, 255 = white) with the values in between representing shades of gray. Vertical bars indicate ± SD. One–way ANOVA with the Duncan’s *post hoc* test was used to determine the significance of differences. Mean values with different letters (a–d) are significantly different (*p* < 0.05, *n* = 5).

The NBT histostains demonstrate that during incubation of seeds in presence of water for 8 h, content of O_2_^•−^ was increased in coleorhiza and scutellum 1.5 times in comparison to the same parts of embryo from dry seeds (Figure 5a,b). During further incubation up to 16 h, O_2_^•−^ showed about 2.5 and 3 times higher level in coleorhiza and scutellum, respectively, than in coleorhiza and scutellum from dry embryo. The signal of O_2_^•−^ was fully suppressed when the seeds were incubated in presence of DPI for 8 and 16 h. After 8 h of imbibition in water, the amount of H_2_O_2_ was ca. 1.7 times higher in coleorhiza than in coleorhiza from dry embryo and remained constant up to 16 h (Figure 5c). The signal of H_2_O_2_ was fully suppressed when the seeds were incubated in presence of CAT for 8 and 16 h.

### 2.4. O_2_^•−^ and H_2_O_2_ Quantification by Flow Cytometry

**Figure 6:**
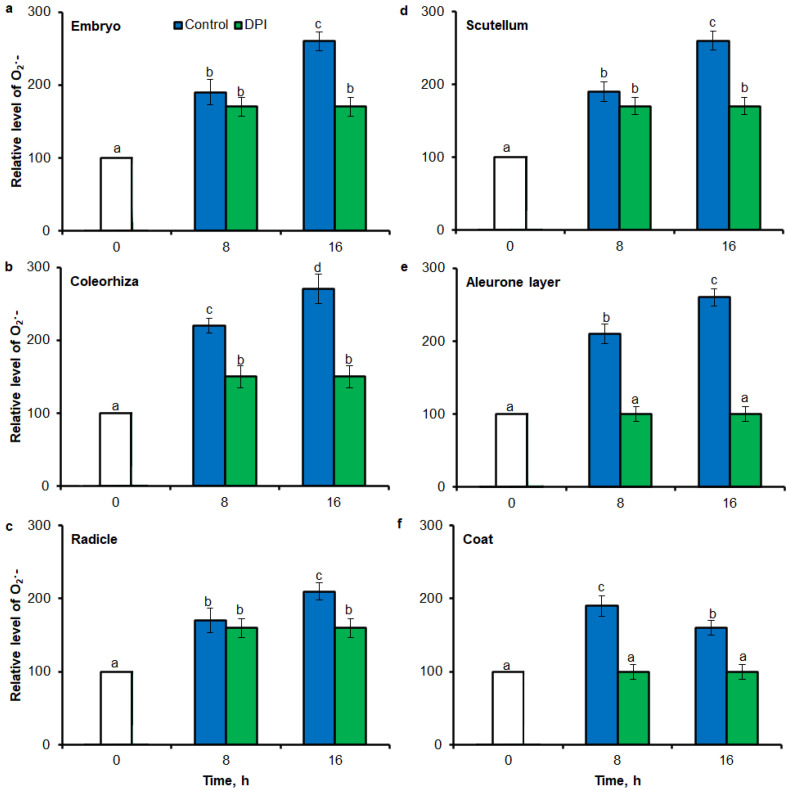
Quantification of O_2_^•−^ in *A. fatua* embryo (**a**), coleorhiza (**b**), radicle (**c**), scutellum (**d**), aleurone layer (**e**) and coat (**f**) of seeds incubated in water or DPI (10^−4^ M) at 20 °C for different time by flow cytometry (FCM). Fluorescence data are expressed as mean fluorescence intensity (percentage of control). No autofluorescence was present when samples were incubated without dye. Vertical bars indicate ± SD. One-way ANOVA with the Duncan’s *post hoc* test was used to determine the significance of differences. Mean values with different letters (a–d) are significantly different (*p* < 0.05, *n* = 5).

Superoxide anion was detected in different parts of seed by flow cytometry (FCM) using the dihydroethdium (DHE), which is oxidized by superoxide to 2-hydroxyethidium. The fluorescence signal showed that after incubation of seeds in presence of water for 8 h, the content of O_2_^•−^ was increased in embryo (Figure 6a), coleorhiza (Figure 6b), radicle (Figure 6c), scutellum (Figure 6d), aleurone layer (Figure 6e) and coat (Figure 6f) of seeds about 1.5 times in comparison to the fluorescence signal from dry seeds. The intensity of the signal increased during the imbibition and after 16 h content of O_2_^•−^ was about 2-fold higher compared with the dry seeds. The content of O_2_^•−^ in coat was similar over the time of imbibition. The detection of O_2_^•−^ was suppressed when the seeds were incubated in presence of DPI; the content of O_2_^•−^ has not changed throughout the whole period of imbibition.

**Figure 7:**
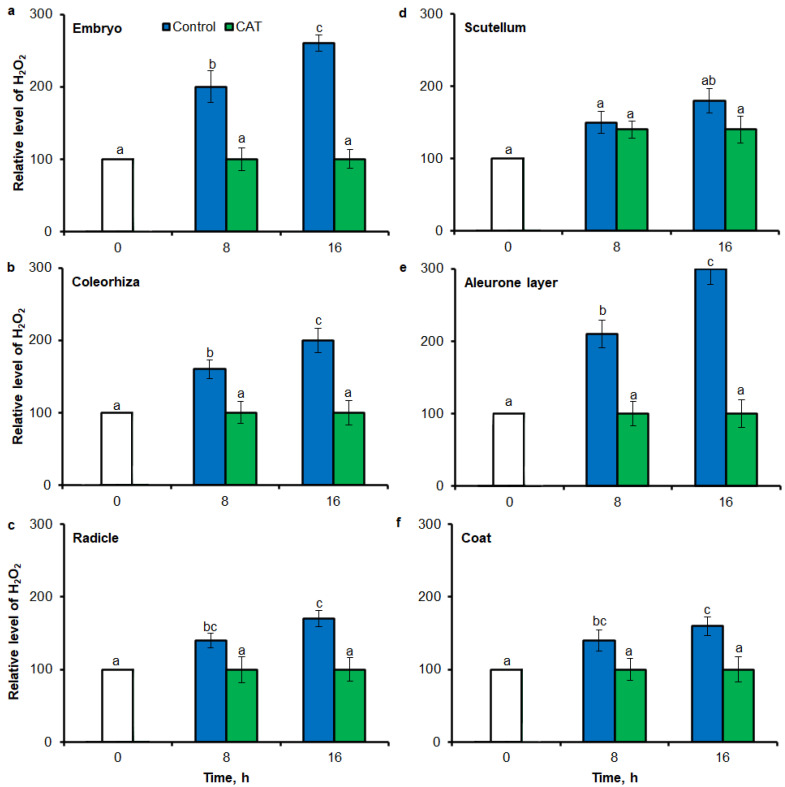
Quantification of H_2_O_2_ in *A. fatua* embryo (**a**), coleorhiza (**b**), radicle (**c**), scutellum (**d**), aleurone layer (**e**) and coat (**f**) of seeds incubated in water or CAT (1000 U) at 20 °C for different time by FCM. Fluorescence data are expressed as mean fluorescence intensity (percentage of control). No autofluorescence was present when samples were incubated without dye. Vertical bars indicate ± SD. One-way ANOVA with the Duncan’s *post hoc* test was used to determine the significance of differences. Mean values with different letters (a–c) are significantly different (*p* < 0.05, *n* = 5).

Fluorescence-based signal for H_2_O_2_ was also detected in different part of seed by flow cytometry (FCM), when seeds were stained using 6-carboxy-2′,7′-dichlorodihydrofluorescein diacetate di(acetoxymethyl ester) (CDCDHFDA-AM). The fluorescence signal was more strongly in embryo (Figure 7a), coleorhiza (Figure 7b), radicle (Figure 7c), aleurone layer (Figure 7e) and coat (Figure 7f) of seeds after incubation for 8 h in comparison to signal from dry seeds; in all parts content of H_2_O_2_ was about 1.5-fold higher. Prolongation of incubation for up to 16 h progressively increased the intensity of the signal; the signal was ca. 1.8-fold higher in embryo, coleorhiza, radicle and aleurone layer from 16 h-imbibed seeds than signal from the dry seeds. Furthermore, in this time point, intensity of the staining in scutellum was only 1.3-fold higher than in scutellum from dry seeds (Figure 7d). The signal of H_2_O_2_ was not detected when the seeds were incubated in presence of CAT for 8 and 16 h.

### 2.5. O_2_^•−^ and H_2_O_2_ Quantification by Spectrophotometric Method

**Figure 8:**
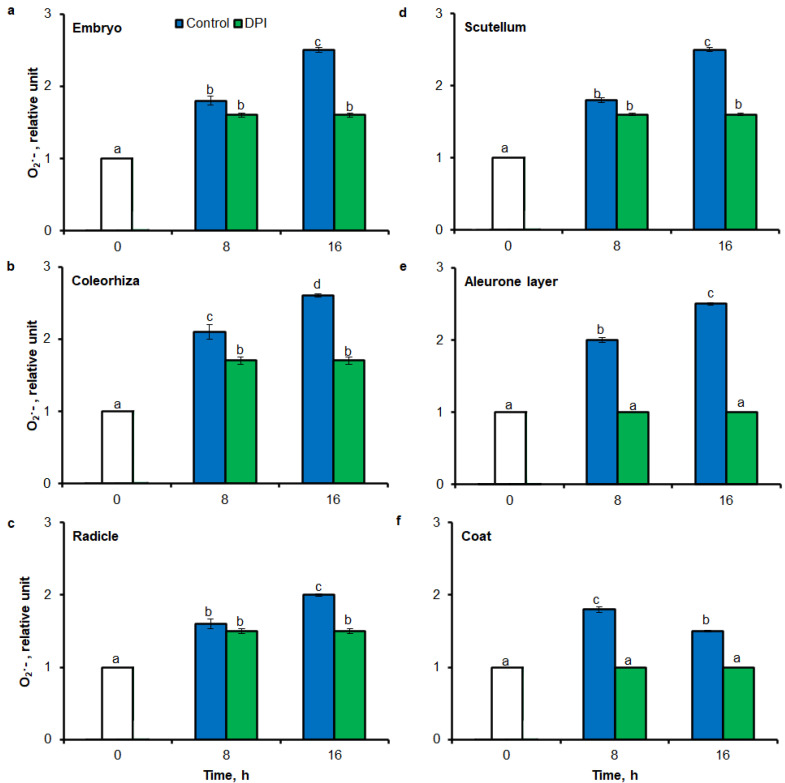
Quantification of O_2_^•−^ in *A. fatua* embryo (**a**), coleorhiza (**b**), radicle (**c**), scutellum (**d**), aleurone layer (**e**) and coat (**f**) of seeds incubated in water or DPI (10^−4^ M) at 20 °C for different time by spectrophotometric method. Vertical bars indicate ± SD. One-way ANOVA with the Duncan’s *post hoc* test was used to determine the significance of differences. Mean values with different letters (a–d) are significantly different (*p*< 0.05, *n* = 5).

To test the precision of tissue printing method with ImageJ and FCM to ROS estimation, intensity of tissue printing signal and stained embryo signal to results obtained using the UV-VIS method were compared. In agreement with higher accumulation of O_2_^•−^ estimated by tissue printing (Figure 1 and Figure 2) and FCM (Figure 6), after 8 h of imbibition in water, the amount of O_2_^•−^ was increased in embryo (Figure 8a), coleorhiza (Figure 8b), radicle (Figure 8c), scutellum (Figure 8d), aleurone layer (Figure 8e) and coat (Figure 8f) of seeds 1.8–2 times in comparison to O_2_^•−^ level in the same parts of dry seeds. After 16 h content of O_2_^•−^ was about 1.8–2.6-fold higher compared with the dry seeds. Moreover, the content of O_2_^•−^ was decreased when the seeds were incubated in presence of DPI; the content of O_2_^•−^ has not changed throughout the whole period of imbibition.

**Figure 9:**
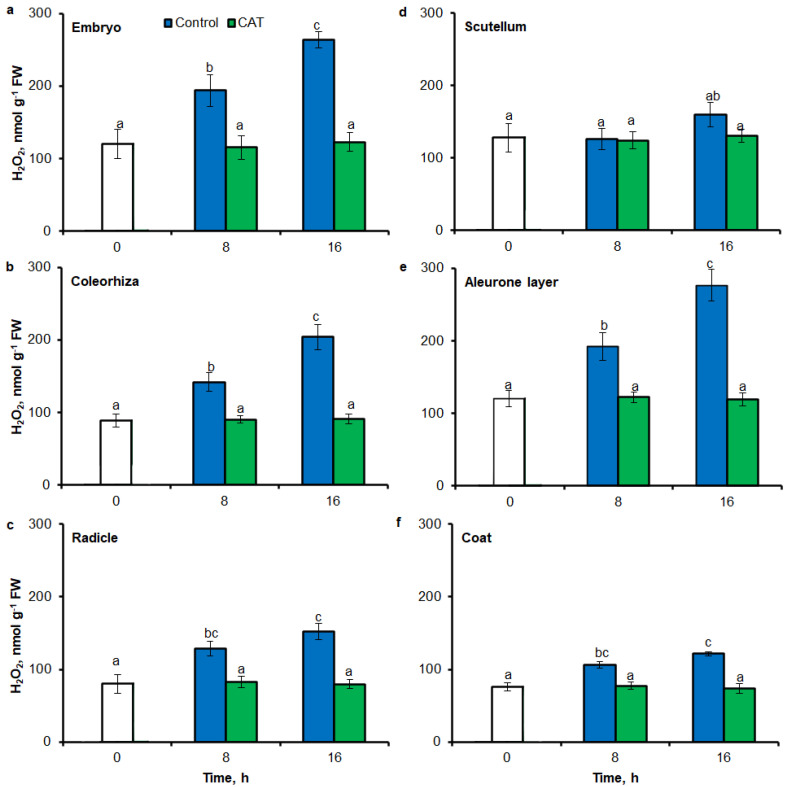
Quantification of H_2_O_2_ in *A. fatua* embryo (**a**), coleorhiza (**b**), radicle (**c**), scutellum (**d**), aleurone layer (**e**) and coat (**f**) of seeds incubated in water or CAT (1000 U) at 20 °C for different time by spectrophotometric method. Vertical bars indicate ± SD. One-way ANOVA with the Duncan’s *post hoc* test was used to determine the significance of differences. Mean values with different letters (a–c) are significantly different (*p* < 0.05, *n* = 5).

After 8 h of incubation in water, level of H_2_O_2_ was increased in embryo (Figure 9a), coleorhiza (Figure 9b), radicle (Figure 9c), aleurone layer (Figure 9e) and coat (Figure 9f) of seeds; after this time content of H_2_O_2_ was about 1.6-fold higher than content of H_2_O_2_ in the same parts of dry seeds. Prolongation of incubation up to 16 h increased the content of H_2_O_2_; in this time point, H_2_O_2_ showed about 1.7–2.2 times higher level than in dry seeds. Furthermore, the level of H_2_O_2_ in scutellum was not significantly changed throughout the whole period of imbibition (Figure 9d). The content of H_2_O_2_ was decreased when the seeds were incubated in presence of CAT.

## 3. Discussion

Seed germination is a critical developmental step regulated by multiple plant endogenous signals, such as phytohormones, reactive oxygen species (ROS) or reactive nitrogen species (RNS) [49,50,51]. ROS are efficiently interlinked with the environmental condition and major hormonal regulators, such as gibberellin, abscisic acid and ethylene, which are associated with seed dormancy and germination [52,53,54]. A number of studies have shown that ROS promote the germination of several seeds, both dormant and non–dormant [6,7,8], including *Avena fatua* [36,55].

Endogenously produced ROS acts as a cell signaling molecules and as a redox potential regulators. However, despite their positive role, their accumulation in the cells leads to oxidative stress. Therefore, understanding the role of ROS as cellular messenger and response for the stress, requires its precise localization, quantification and global dynamics of ROS in different parts of the seed. Due to short half-life and high reactivity, the detection of ROS in the cell has always been very challenging. During past decades, a variety of methods for detection and quantification of ROS, including its reactive intermediates, have been applied.

Tissue printing method has become an important tool for visualization and localization of different molecules in plant tissue [56]. In this study, it has been used to detect and determine the localization of ROS in non-dormant *Avena fatua* L. seeds during imbibition. One of known techniques of detection of O_2_^•−^ is histochemical staining based on the reduction of nitro blue tetrazolium by O_2_^•^, visualized by the formation of the dark blue formazan [18]. Employment of this reaction together with the tissue printing method, made the detection and localization of O_2_^•−^ in plant tissue possible. According to our knowledge, the detection and localization of O_2_^•−^ using the NBT-mediated tissue printing in plant material is unavailable. In our protocol, O_2_^•−^ was localized in embryo, coleorhiza, radicle, scutellum, aleurone layer and coat during seeds imbibition up to 16 h (Figure 1 and Figure 2). However, when the seeds were incubated in presence of DPI, intensity of staining was completely decreased. Until now, the tissue printing for H_2_O_2_ detection and localization was used only for seedlings of *Glycine max*, *Pisum sativum*, *Phaseolus vulgaris*, *Helianthus annuus*, *Cucumis sativus* and *Solanum tuberosum* [29]. It is worth noting, that aforementioned procedure was based on the oxidation of KI to I_2_ by H_2_O_2_. Used nitrocellulose membranes were presoaked with starch and KI, reagent mixture for the histochemical assay of H_2_O_2_ [57]. The DAB–mediated tissue printing, has been proposed for direct detection of H_2_O_2_ production also for seedlings of *Zea mays* [30] and *Lycopersicon esculentum* [31], as well as fruits of *L. esculentum* [32]. Usage of this method allowed for identification of H_2_O_2_ signals in coleorhiza and radicle of embryo, aleurone layer and coat during seeds imbibition up to 16 h (Figure 1 and Figure 4). Nonetheless, when the seeds were incubated in presence of CAT, intensity of staining was entirely decreased. ROS accumulation in coleorhiza and radicle can be associated with elongation growth and cell wall loosening. Previously, ROS have been demonstrated to play a role in radicle elongation in *Lepidium sativum* [58] and *Lactuca sativa* [33].

Taken as a whole, presented results show that NBT- or DAB-mediated tissue printing for O_2_^•−^ and H_2_O_2_, respectively, are a very rapid and specific methods which can be used for detection and visualization of ROS in seeds. Rapidity of this protocol (within 10 s, see Materials and Methods for details) can completely avoid the interference of wound-induced ROS.

Usage of image processing software for further analysis of received results, is fundamental and allow to extract very useful information from images. Results analysis can be done using open source image analysis tools, such as CellProfiler [59], Fiji/ImageJ [60] or Icy [61] can be used. ImageJ, a Java-based application, currently very popular in human science, thanks to its versatility can also be used as a tool for the quantification of histological results [62]. In the field of plant and seed science, ImageJ provides a way to measure many parameters, such as length, width, area or shape [63] and has become necessary tool for plant physiology, such as leaf disease or leaf color changes [64].

There are several example studies where the image processing method was used for the quantitative estimate of ROS accumulation in leaves. ImageJ has been used for determination of O_2_^•−^ and H_2_O_2_ level in leaves [41,42,43,65] and roots [66]. According to available data, O_2_^•−^ and H_2_O_2_ in seeds are visualized by staining with NBT or DAB, respectively. The data interpretation was based on subjective visual estimation and provided only qualitative results. Stained tissue can be digitalized and opened in Fiji/ImageJ for quantification of blue (NBT for O_2_^•−^) or yellow (DAB for H_2_O_2_) color intensity (in the negative, corresponding to the lighter tones of gray). In this work, aforementioned method has been applied for NBT- or DAB-mediated tissue printing of whole seeds and histochemical stained embryo, as well as used to detect the spatial distribution of the NBT or DAB intensity at different parts of seeds. As a result, an image with NBT or DAB only staining is generated and the average intensity of its pixels can be quantified after the selection of specific ROI (*Region of Interest*). In digital image analysis, the pixel intensity values for color range from 0 to 255, wherein, 0 represents the darkest shade of the color and 255 represent the lightest shade of the color as a standard. Combining tissue printing and image processing with Fiji/ImageJ software, it was possible to obtain a relative ROS concentration based on the pixel intensity resulting from the NBT or DAB signal (Figure 1, Figure 2, Figure 3, Figure 4 and Figure 5). Obtained results demonstrate that the Fiji/ImageJ is a promising tool for the quantification of ROS in different part of seeds.

Oxidative stress can be studied using a large variety of methodological approaches, including fluorescence: microscopy or classical fluorimeter. Several fluorescent dyes have been tested so far. They allowed to obtain information on the localization of ROS and thus the dynamics of oxidative stress in plants exposed to unfavorable environmental conditions. In present study, small–molecule fluorescent ROS dyes (DHE and CDCDHFDA-AM) and dual excitation flow cytometry, which can provide information at the single cell level, have been used for ROS quantification in different parts of seeds. The fluorescence signal showed that during imbibition of seeds up to 16 h, the content of O_2_^•−^ was increased in embryo (Figure 6a), coleorhiza (Figure 6b), radicle (Figure 6c), scutellum (Figure 6d), aleurone layer (Figure 6e) and coat (Figure 6f). The fluorescence signal also for H_2_O_2_ was more strongly in embryo (Figure 7a), coleorhiza (Figure 7b), radicle (Figure 7c), aleurone layer (Figure 7e) and coat (Figure 7f). To confirm that FCM measures the ROS level, caryopses were treated with DPI or CAT. The application of DPI or CAT, fully reduced of fluorescence signal intensity.

In plant physiology, the fluorescence probes DHE and CDCDHFDA-AM are perhaps the most frequently used when studying ROS signaling and oxidative stress in plant cells and green algae using fluorescence microscopy and fluorometry [67]. To our knowledge, the present study is the first application of dual excitation FCM with DHE and CDCDHFDA-AM to determination of O_2_^•-^ and H_2_O_2_ level in seeds. The results obtained demonstrate that FCM is a promising tool for the precise localization and quantification of ROS in different part of seeds.

To validate results obtained using tissue printing with image analysis and FCM methods for ROS analysis in seed material, UV-VIS method was used to measure O_2_^•−^ and H_2_O_2_ levels. In this study, it was proved a high correlation between mean gray value in tissue printing signal, FCM signal and the biochemically—quantified amount of O_2_^•−^ and H_2_O_2_ (Figure 8 and Figure 9). In agreement with higher accumulation of O_2_^•−^ estimated by tissue printing (Figure 1, Figure 2 and Figure 3) and FCM (Figure 6 and Figure 7), during imbibition up to 16 h, the amount of O_2_^•−^ and H_2_O_2_ was increased in embryo (Figure 8a and Figure 9a), coleorhiza (Figure 8b and Figure 9b), radicle (Figure 8c and Figure 9c), aleurone layer (Figure 8e and Figure 9e) and coat (Figure 8f and Figure 9f). Furthermore, the level of O_2_^•−^ increased in scutellum (Figure 8d) but the level of H_2_O_2_ was not significantly changed in scutellum throughout the whole period of imbibition (Figure 9d). The content of O_2_^•−^ and H_2_O_2_ was decreased when the seeds were incubated in presence of DPI or CAT, respectively.

## 4. Materials and Methods

### 4.1. Seed Material

*Avena fatua* (wild oat) spikelets, which contained 2–3 florets covered with glumes, were collected in July 2010 near Szczecin (Poland). After collection, the florets (a single caryopsis, covered by the lemma and palea) were dried at room temperature (RT) for 7 days to a constant moisture content (ca. 12%). To obtain non–dormant caryopses (seeds), dormant florets were stored dry under ambient relative humidity for up to 4 months in darkness at 25 °C.

In all the experiments, seeds (25 in each of 5 replicates) were incubated at 20 °C in the dark, in Petri dishes (ø 6 cm) on one layer of filter paper (Whatman No. 1) moistened with 1.5 mL of distilled water or with solutions: diphenyleneiodonium (DPI) (10^−4^ M) (Merck, Darmstadt, Germany) or catalase (CAT) (1000 U) (Merck, Darmstadt, Germany) for 8 or 16 h. After treatment whole seeds and embryo, coleorhiza, radicle, coat (testa + pericarp) and aleurone layer isolated from caryopses were used for analysis.

### 4.2. O_2_^•−^ and H_2_O_2_ Localization by NBT- and DAB-Mediated Tissue Printing and Content Determination by ImageJ

Localization of O_2_^•−^ and H_2_O_2_ using tissue printing was determined according to Liu et al. [31] with some modification. After incubation whole seeds were placed on Petri dish and cut using razor blade. To obtain a high—quality cut surface without damaging tissue, each edge of the razor blade was used several times. Longitudinally bisected half seed was immediately used for tissue printing. Nitrocellulose blotting membrane (Amersham™ Protran™ 0.2 μm NC, Amersham, UK) was soaked in 6 mM NBT (Merck, Darmstadt, Germany) (in 10 mM Tris-HCl, pH 7.4) for O_2_^•−^ or 2 mg ml^−1^ DAB-HCl solution (pH 3.8) (Merck, Darmstadt, Germany) for H_2_O_2_ localization and then air-dried at 25 °C for 30 min in darkness. The soaked membrane was placed on three layers of filter paper (Whatman No 1) (Merck, Darmstadt, Germany). The half seed was placed with its cut surface down on membrane, covered with parafilm and pressed for 10 s. Then, the half seed or embryo was carefully removed with the forceps. After 5 min at RT, when reaction between ROS and NBT or DAB was completed, membranes were photographed (Canon EOS 500, Tokyo, Japan). All the images were saved as TIFF files, with 3072 × 2304 pixel resolution and 24 bits RGB color depth (pixel transformation factor = 1, no scaling of result images). The image analysis was conducted using the ImageJ (version 1.51s/Java 1.6, LOCI, University of Wisconsin, U.S.). NBT or DAB stained membranes were converted to gray scale for analysis. Staining can be assessed by setting a ‘threshold’ using the thresholding tool. However, to limit the ROS determination to the defined parts of the seeds or embryos, specific ROI (*Region of Interest*; using one of the drawing tools) was analyzed. Minimum and maximum threshold values were established to remove background staining and a mean gray value for the plaques was then calculated. In digital image analysis, the pixel intensity values for color range from 0 to 255, wherein, 0 represents the darkest shade of the color and 255 represent the lightest shade of the color as a standard. The data are presented as a mean gray value of the total plaque area to give a relative staining density for each sample [68]. The dried membranes can be kept at room temperature without losing signals or the original resolution of printed images for several months.

To verify whether there is a production of wound-induced ROS under experimental conditions, tissue printing was performed at 0, 1, 2 and 4 min after cutting.

### 4.3. In Situ Localization and Level of O_2_^•−^ and H_2_O_2_ and Content Determination by ImageJ

Generation of ROS in situ was detected by monitoring the reduction of nitro blue tetrazolium (NBT) as described by Beyer and Fridovich [18] or polymerization of 3,3′–diaminobenzidine (DAB) according to Thordal-Christensen et al. [19]. After incubation embryos were isolated from seeds and stained for 10 min in darkness in 6 mM NBT (in 10 mM Tris-HCl, pH 7.4) or 90 min in darkness in 1 mg ml-1 DAB containing 0.05% (*v*/*v*) Tween-20 and 10 mM Na_2_HPO_4_. Dark blue staining in the presence of NBT indicated O_2_^•−^ production or yellow staining in the presence of DAB indicated polymerization of DAB, requiring H_2_O_2_ and peroxidase activity. After removing the staining solution and rinsed embryo three times in sterile water, embryos were placed on a highly saturated color background, different from color of stained area and photographed (Canon EOS 500). The image analysis was conducted using the ImageJ.

### 4.4. Determination of O_2_^•−^ and H_2_O_2_ Content by Flow Cytometry

To measure the amount of ROS generated by seeds, a membrane-permeable form of dye, dihydroethdium (DHE) (ThermoFisher, Waltham, MA, USA) for O_2_^•−^ and CDCDHFDA-AM (6-carboxy-2′,7′-dichlorodihydrofluorescein diacetate) (ThermoFisher, Waltham, MA, USA) for H_2_O_2_, were used. After incubation, the embryos, coleorhiza, radicle, scutellum, coat (testa + pericarp) and aleurone layer were isolated from seeds. Sample were incubated in 20 µM DHE or CDCDHFDA-AM in 0.1 M K_2_HPO_4_/KH_2_PO_4_ pH 7.4 for 60 min at 25 °C in the dark. After incubation, samples were washed three times in 0.1 M K_2_HPO_4_/KH_2_PO_4_ pH 7.4.

The stained samples were transferred to 2 mL of the isolation buffer, pH 7.4, containing 45 mM MgCl_2_, 30 mM sodium citrate, 20 mM 3-(*N*-morpholino)propanesulfonic acid (MOPS) and 0.1% (*v*/*v*) Triton X-100 [69] and using a razor blade, were chopped for 2 min. Subsequently, the suspension was passed through a 50 µm nylon mesh. The labelled cells were analyzed using flow cytometer (Partec, Germany) with an air-cooled 20 mV argon-ion laser and HBO mercury arc lamp. In the flow chamber, each cell crosses a region of fluorescence excitation. There, the green- or red-fluorescence emissions are excited consecutively with blue light at 488 nm and UV and recorded by photomultiplier tubes. The flow cytometer was equipped with dichroic mirror with the edge at 420 nm (TK420), a full mirror (FM) and a long pass filter with the edge at 515 nm (OG515). The final gated cell populations contained 20,000 cells and signals were recorded on a histogram by logarithmic amplifiers. The histograms present the fluorescence intensity (log; Geo Mean) on the *x*-axis and cell count on the *y*-axis in gated population of cells. The relative O_2_^•−^ and H_2_O_2_ level was expressed as the mean fluorescence intensity (percentage of the control).

### 4.5. Determination of O_2_^•−^ and H_2_O_2_ Content by Spectrophotometric Method

Extracellular O_2_^•−^ production was estimated using the method developed by Misra and Fridovich [17]. After incubation, the embryos, coleorhiza, radicle, scutellum, coat (testa + pericarp) and aleurone layer were isolated from seeds. Sample were ground and homogenized by pestle and mortar in cold 50 mM Tris-HCl pH 7.5 with 2% (*w*/*v*) PVPP (fresh weight: buffer, 1:10, *w*/*v*). The homogenate was centrifuged for 20 min at 14,000× *g* at 4 °C and then the supernatant was immediately used. The reaction mixture was composed of 0.05 mL of 50 mM Tris-HCl pH 7.5, 0.05 mL of 60 mM epinephrine (in 0.5 M HCl) and 0.05 mL of supernatant. The oxidation of epinephrine to adrenochrome was measured in reaction mixture at 480 nm for 2 min. In each test, oxidation of adrenaline was carried out in the reaction mixture (without extract). The epinephrine extinction coefficient was ε = 4.02 mM^−1^ cm^−1^. The results were expressed as relative unit corresponds to the rate of epinephrine oxidation in extracts of sample from dry caryopses calculated as μmol min^−1^ g^−1^ FW).

Extracellular H_2_O_2_ content was determined according to Velikova et al. [70]. After incubation, the embryos, coleorhiza, radicle, scutellum, coat (testa + pericarp) and aleurone layer were isolated from seeds. Sample were ground and homogenized by pestle and mortar in cold 1% (*w*/*v*) trichloroacetic acid (TCA) (fresh weight:TCA, 1:10, *w*/*v*). After 20 min of centrifugation at 14,000× *g* at 4 °C, the resulting supernatant was immediately used for spectrophotometric analysis. The content of H_2_O_2_ was measured in reaction mixture (0.5 mL of 10 mM potassium phosphate buffer pH 7.0, 1 mL of 1 M KI in 10 mM potassium phosphate buffer, pH 7.0 and 0.5 mL of the supernatant) at 390 nm after incubation at 25 °C for 60 min. A standard curve was prepared by using the H_2_O_2_ standard. The results were expressed as nmol H_2_O_2_ g^−1^ FW.

### 4.6. Data Analysis

All the experiments were carried out in five biological replicates and the results are expressed as mean ± SD. The means were analyzed for significance using one-way analysis of variance, ANOVA (Statistica for Windows version 13.0, Stat-Soft Inc., Tulsa, OK, USA). Data were checked for normality and homogeneity of variance and met these criteria. Duncan’s multiple range test was used to test for significance of differences (*p* ≤ 0.05). Every experiment was repeated three times and the results presented correspond to a representative single experiment.

## 5. Conclusions

ROS are hot topics in seed biology because they function both as agents of damage and mediators of cellular signals. This small molecules are now known to play an important role in seed dormancy and germination, stress responses and environmental interactions. Understanding of their functions needs knowledge about the level of ROS, especially superoxide anion and hydrogen peroxide. Hence, it is necessary to use sensitive and specific methods in order to understand the contribution of each signaling molecule to various biological processes.

The quantitative method for ROS determination based on tissue printing with image analysis and flow cytometry are simple and fast. This methods can be used for determination of ROS accumulation in the external and inner parts of the seeds. Finally, all steps of tissue printing protocol was done within 10 s which avoided interference of ROS resulting of tissue damage. Image analysis applied to NBT- or DAB-mediated tissue printing and FCM provides a very efficient means of quantifies ROS level in seed sample. It has been practically proven, by comparison of received results with the UV-VIS results, that developed methods provide repeatable, accurate and reliable results. Furthermore, the use of image analysis methods avoids the effects of human subjectivity. These methods have the potential to significantly enhance both precision and reproducibility and making quantitative methods more readily accessible to most plant laboratories.

## Supplementary Materials

The following are available online at https://www.mdpi.com/1422-0067/21/22/8656/s1, Figure S1: Detection and localization of O_2_•− and H_2_O_2_ in *Avena fatua* seeds incubated in water at 20 °C for 8 or 16 h by NBT– or DAB–mediated tissue printing of seeds at 4 min after cutting (representative tissue printing are shown); Figure S2: In situ localization of O_2_•− (a, b) and H_2_O_2_ (c, d) in *A. fatua* seeds incubated in water at 20 °C for 8 or 16 h. After staining whole seeds were cut using razor blade (b, d). Representative stained seeds are shown; Figure S3: In situ localization of O_2_•− and H_2_O_2_ in *A. fatua* seeds incubated in water at 20 °C for 8 or 16 h. After incubation of seeds, longitudinally bisected half seeds were stained. Representative stained seeds are shown.

## Abbreviations

CDCDHFDA	6-carboxy-2′,7′-dichlorodihydrofluorescein diacetate di(acetoxymethyl ester)
DAB	3,3′-diaminobenzidine
DHE	dihydroethidium
DPI	Diphenyleneiodonium
FCM	Flow cytometry
NADPH oxidase	Nicotinamide adenine dinucleotide phosphate oxidase
NBT	Nitro blue tetrazolium
PCD	Programmed cell death
ROS	Reactive Oxygen Species
