# Supplementary material for: Tissue Printing and Dual Excitation Flow Cytometry for Oxidative Stress—New Tools for Reactive Oxygen Species Research in Seed Biology
Source: Int J Mol Sci. 2020 Nov 17;21(22):8656. doi: 10.3390/ijms21228656 (PMC7697308; doi:10.3390/ijms21228656)

Tissue Printing and Dual Excitation Flow Cytometry for Oxidative Stress—New Tools for Reactive Oxygen Species Research in Seed Biology

Danuta Cembrowska-Lech

Institute of Biology, University of Szczecin, Wąska 13, 71-415 Szczecin, Poland; danuta.cembrowska-lech@usz.edu.pl

Supplementary data

Figure S1. Detection and localization of $O_2{\bullet}^-$ and H_2O_2 in *Avena fatua* seeds incubated in water at 20 °C for 8 or 16 h by NBT– or DAB–mediated tissue printing of seeds at 4 min after cutting (representative tissue printing are shown).

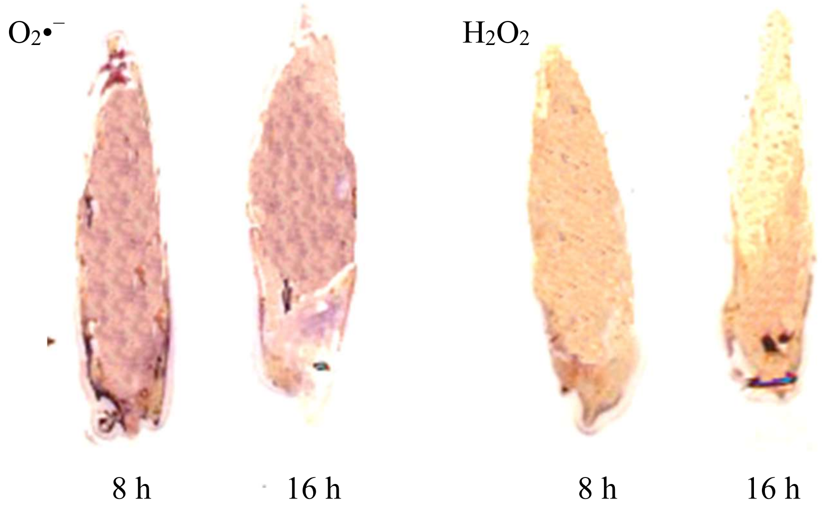

Figure S2. *In situ* localization of $O_2{\bullet}^-$ (a, b) and H_2O_2 (c, d) in *A. fatua* seeds incubated in water at 20 °C for 8 or 16 h. After staining whole seeds were cut using razor blade (b, d). Representative stained seeds are shown.

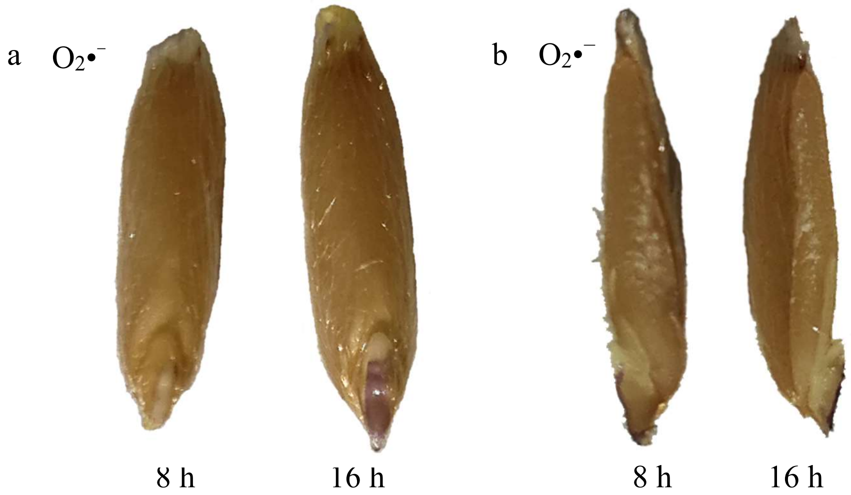

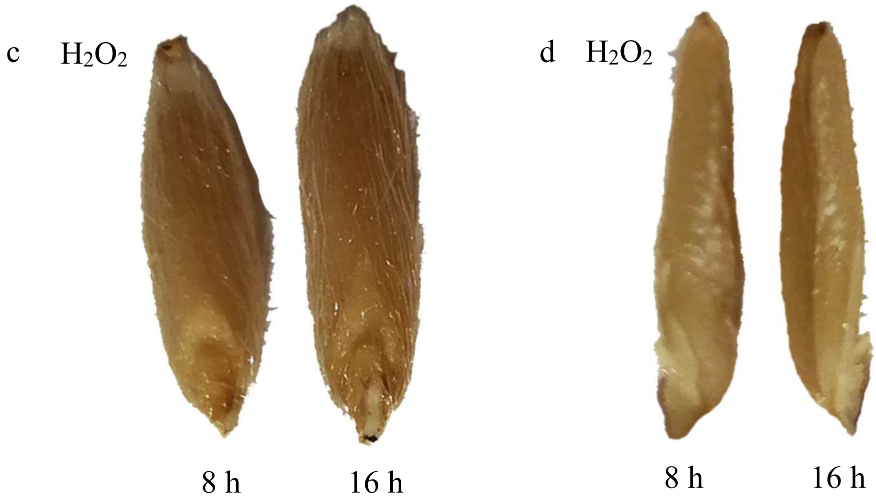

Figure S3. *In situ* localization of $O_2{\bullet}^-$ and H_2O_2 in *A. fatua* seeds incubated in water at 20 °C for 8 or 16 h. After incubation of seeds, longitudinally bisected half seeds were stained. Representative stained seeds are shown.

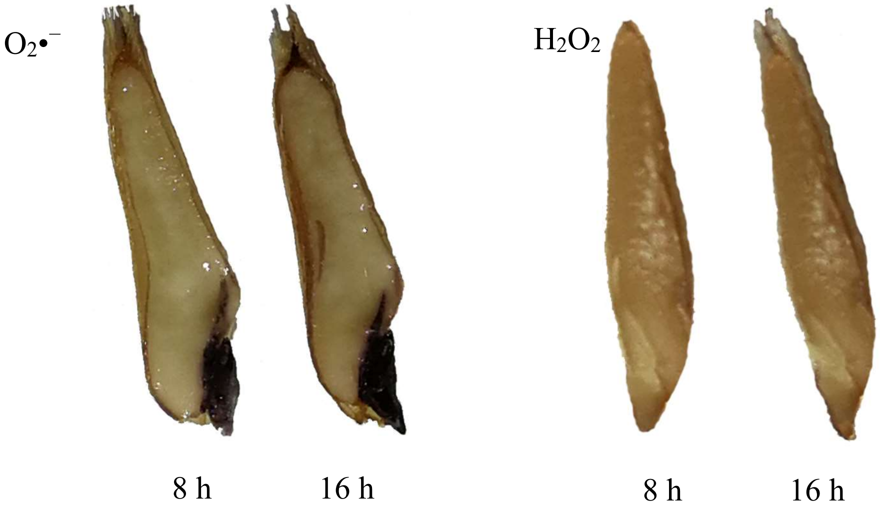

Supplement: Supplementary file 1 [file ijms-21-08656-s001.pdf]
